# Out of sight out of mind: Psychological distance and opinion about the age of penal majority

**DOI:** 10.3389/fpsyg.2022.763335

**Published:** 2022-09-15

**Authors:** Ivete Furtado Ribeiro Caldas, Igor de Moraes Paim, Karla Tereza Figueiredo Leite, Harold Dias de Mello Junior, Patrícia Unger Raphael Bataglia, Raul Aragão Martins, Antonio Pereira

**Affiliations:** ^1^Department of Morphology and Physiological Sciences, Pará State University, Marabá, Brazil; ^2^Federal Institute of Education, Science and Technology of Ceará, Maranguape, Brazil; ^3^Department of Electrical Engineering, Faculty of Engineering, Rio de Janeiro State University, Rio de Janeiro, Brazil; ^4^Department of Education and Human Development, Paulista State University, Marilia, Brazil; ^5^Department of Education, Paulista State University, Sao Jose do Rio Preto, Brazil; ^6^Department of Electrical and Biomedical Engineering, Institute of Technology, Federal University of Pará, Belem, Brazil

**Keywords:** psychological distance, moral development, public opinion, youth justice, adolescence

## Abstract

The growth of urban violence in Brazil, as in other countries, has led citizens to demand more severe and punitive measures to solve the problem of juvenile crime. One motion submitted to the Brazilian parliament, for instance, proposes to reduce the age of penal majority (APM) from 18 to 16 years. Our hypothesis is that popular opinions about this proposal are largely constrained by construal levels and psychological distance. Accordingly, we expect that the knowledge and proximity to the circumstances associated with juvenile transgression will influence opinions about the proposal. To test this hypothesis, we evaluated how opinion against or for the proposal can be explained by psychological distance and moral development theory. We studied two samples, composed of people who do not have a deep experience with the subject (passersby in a public square (*N* = 77) and workers from a juvenile justice court (*N* = 157). After collecting socio-demographic information from the subjects and their answer to moral dilemmas, the data was subjected to a multivariate analysis by multimodal logistic regression for socio-demographic characteristics, Kohlberg moral stages, and opinion on the reduction of APM (agree, indifferent, and disagree) as dependent variables. Our findings suggest that 1) opinion about the APM depends on psychological distance and 2) socioeconomic variables may influence the average construal level of adolescent transgressors in the public’s perspective.

## Introduction

The understanding of adolescence as a distinct phase of development interposed between the more recognizable stages of childhood and adulthood has been a dilemma to many cultures throughout the ages (Sawyer et al., 2018). In primitive societies, signs of puberty triggered “rites of passage” marking the transition for children into adulthood (Alcorta and Sosis, 2020). In those early hunter-gatherer groups and subsequent human generations, there was increased expectation that adolescents quickly obtained adult-level subsistence skills and contributed more effectively to their communities. Therefore, there was a great effort from the group’s elders to teach children complex skills, such as hunting, manufacturing tools, and preparing food (Lew-Levy et al., 2017).

External signs of puberty are initiated by the activation of the neuroendocrine hypothalamic–pituitary–gonadal axis that induces robust increases in gonadotropins, which, by their turn, stimulate the gonads, ovary and testis, to develop and produce the sex steroids estrogens and androgens, respectively, and trigger the development of secondary sex characteristics (Delemarre et al., 2008). However, the dynamics between the socio-cultural perception of the outward signs of puberty and adolescent behavior are not perfectly juxtaposed (Worthman and Trang, 2018). For instance, while society expects adolescents to quickly demonstrate adult-like attitudes the most characteristic signs of this phase of development is an increase of risky behavior and low resistance to peer influence (Albert et al., 2013; Casey et al., 2020). As a result of this developmental constraint, adolescents, when compared to other age groups, are more likely to engage in transgressive experiments with addictive substances, violent and non-violent crime, and careless driving (Zimring, 2000), thus increasing their chances of conflict with the law. In western populations, the prevalence of criminal behavior increases from late childhood, peak during adolescence and then declines in adulthood, forming a bell-shaped age–crime curve (Moffitt, 2018). However, most violent crimes, such as homicides, are more frequently committed by adults. For instance, in the United States in 2020, more than 92% of murders were committed by individuals older than 18 (FBI, 2020).

As in childhood, optimal development in adolescence is predicated on a synergy between biology and socio-cultural context. Brain development, specifically, is characterized by the existence of distinct critical periods of plasticity during which the maturation of cortical circuits is more susceptible to environmental influence (Hensch, 2005). During adolescence, the most important brain region still in the process of maturation is the prefrontal cortex (PFC) (Paus et al., 1999), which is responsible for higher cognitive functions, including decision-making and emotional control. Also, during adolescence, adult-like connections between the PFC and the amygdala, an important region associated with signaling emotionally or motivationally relevant stimuli to the brain, begin to emerge (Tottenham and Gabard-Durnam, 2017). The association between the immaturity of key cortical areas and pathways associated with decision-making and emotional control and the search for freedom from parental supervision and affiliation to social circles probably underlies the characteristic impulsiveness and rebelliousness of adolescent behavior and is considered part of normal brain maturation (Carlisi et al., 2020). However, negative stereotypes are rife in the adult perceptions of adolescents’ behavior. Even before Hall (1904) characterized the adolescent period as “storm and stress,” youth behavior, associated with impulsiveness and emotional immaturity, has been viewed with reservation by many adults (Altikulaç et al., 2019). Previous works showed that this view is prevalent among both parents and teachers (Buchanan et al., 1990; Hines and Paulson, 2006). Gross and Hardin (2007) showed that stereotypes of adolescents influence explicit evaluations unconsciously and unintentionally.

Modern life has been associated with an increase in the span of the adolescence phase, both through the acceleration of puberty’s arrival (Eckert-Lind et al., 2020) and a rise in the sociocultural thresholds for attaining adulthood (Hochberg and Konner, 2020). Nowadays, adolescence occupies a greater extent of human life course than in earlier periods (Patton and Viner, 2007). Consequently, there is a growing need for an expanded and more inclusive definition of adolescence in both law and social policies. However, society has been slow to catch up on these scientific findings and promote the updating and the appropriate framing of adolescent-related laws. Some initiatives, though, have been implemented and deserve mention, such as the creation in 2015 of a Young Adult Court (YAC) in San Francisco for eligible young adults, ages 18–24, and which proposes to align opportunities for accountability and transformation with the unique needs and developmental stage of this age group (Stamm, 2017).

Penal populism refers to an understanding of justice in which criminal and anti-social or deviant activity should be harshly punished (Pratt, 2007). This doctrine has been very popular due to the recent wave of populist leaders coming to power in many countries (Kenny and Holmes, 2020). One of the key proposals of penal populism is to decrease the age of penal majority (APM) in countries with penal codes they think are “extremely lenient” with juvenile transgressors. The present study was conducted in Brazil, where there are several proposals being discussed in congress to amend the constitution and decrease the APR from 18 to 16 y.o. (Vavassori and Toneli, 2015). First, we will present the problem of adolescent transgression in Brazil and then we will frame our experimental approach which was based on construal level theory and Kohlberg’s theory of moral development.

### Adolescent transgressions

The rapid growth of urban youth violence has increased popular demand for more severe and punitive measures against transgressors. In Brazil, a country with extreme levels of income inequality, the number of adolescents sentenced to socio-educational measures increased about 100% in 1 year, from 96,000 in 2018 to 189,000 in 2019. The discussion of punishment for youth transgression, however, is usually framed by the mistaken perception of adolescents as the main cause of violence rather than as victims, and that existing laws excessively protect juvenile offenders (Brondani and Arpini, 2021). As a result, Brazilian legislators proposed an amendment to the constitution to lower the APM from 18 to 16 years (Vavassori and Toneli, 2015; Petry and Nascimento, 2016). The amendment is currently under consideration in the Senate’s Constitution, Justice, and Citizenship Committee. It specifically proposes the modification of art. 228 of the Federal Constitution so that criminal responsibility moves from 18 to 16 years for general crimes, and from 18 to 14 years for heinous crimes, torture, narcotics’ trafficking, terrorism, and membership in a criminal organization. The most recent national poll from 2019 showed that 84% of the population approve the measure and this level has remained constant over the years since 2013 when it was first proposed (G1, 2019).

Since most juvenile criminal defendants in Brazil come from the lowest socioeconomic echelons of society, this measure would probably combine with a life history marked by neglect and precarious living conditions to trap them in an endless circle of poverty and recidivism (Petry and Nascimento, 2016; Brondani and Arpini, 2021). If the measure is eventually approved, adolescent transgressors could end up being incarcerated in adult penal facilities, which in Brazil are notorious for violence and bad living conditions in general (Constantino et al., 2016; Butler et al., 2018), in the middle of circuit building between the PFC and the amygdala in their brains (Tottenham and Gabard-Durnam, 2017). Recent experimental work from our group has already demonstrated that exposure to chronic stress and impoverished environments can compromise the development of the PFC in adolescent rats (Folha et al., 2017). This finding suggests that the same could occur in humans and the exposure of youth transgressors to the harsh environment conditions of adult prisons holds the risk of stunting their PFC development and compromising their chances of rehabilitation (Casey et al., 2010). While this can be considered a tragic outcome individually, it would be also costly to society in terms of waste of human capital.

### Construal level theory

Social expectations and opinions about adolescence are variable and contingent upon culture. However, there is a widespread tendency to hold negative stereotypes against adolescents, both explicitly and implicitly (Gross and Hardin, 2007). According to Construal Level Theory (CLT), mental representations of persons are based on a continuum from personalized, or concrete, to more abstract, category-based representations, depending on the psychological distance of the perceiver (Trope and Liberman, 2010). We tend to think in concrete ways about entities and events which are spatially, temporally, emotionally, or socially close to us, and in abstract ways about entities and events perceived as distant according to the same parameters (Trope and Liberman, 2010). According to CLT, when judging other people’s behavior, we are more readily inclined to apply our moral principles to psychologically distant than proximate targets (Eyal et al., 2008; Mentovich et al., 2016). An increase in psychological distance minimizes sensitivity to intrinsic characteristics of the targets while focusing on more diffuse factors such as gender, ethnicity, and social class (Mentovich et al., 2016; Yudkin et al., 2016). Thus, greater psychological distance makes us more vulnerable to stereotypes and other cognitive biases.

Stereotyping can lead to systematic misperceptions or misjudgments of reality based on preconceived beliefs, rather than relevant facts and actual enquiry. Commonly held stereotypes about adolescents can thus get in the way of a more reasonable understanding of their actual vulnerability and neglect by society. Unfortunately, the cognitive heuristics underlying such stereotypes are deeply ingrained in the human mind. However, some studies have suggested they can be modified by interventions aimed at changing the degree of abstract and concrete construal mindsets (McCrea et al., 2012).

### Moral development

Humans are distinct from other animals for their deep concern over issues of morality, justice, and fairness (Decety and Cowell, 2018). We are also unique in establishing organizations and institutions to enforce social norms and assign appropriate punishments to violators (Buckholtz and Marois, 2012). Legal systems evolved from the need to organize life in complex human groups and were initially systematized from primitive moral codes.

Human morality arose as a set of skills and motives for cooperating with others and thus promote group welfare (Tomasello and Vaish, 2013). The roots of human morality can be glimpsed in cooperative behaviors seen in many non-human primate groups. Though, different from them, our expectations of what others should do are also guided by shared norms, not only statistical inference. Human morality develops through increasingly complex cognitive rationales for making moral judgments and decisions.

Kohlberg proposed that the development of human morality proceeds through Pre-conventional (stages 1 and 2), Conventional (stages 3 and 4), and Post-Conventional (stages 5 and 6) levels of reasoning, with each of these levels being composed of two stages, thus making a total of six stages (Kohlberg, 1958, 1981, 1984; Turiel, 1966; Rest et al., 1969; Lapsley, 1992). According to Kohlberg, most children have a pre-conventional morality, most adults have a conventional one, and only 20 to 25% of the adult population attains the post-conventional level (Kohlberg, 1974). Kohlberg stages of moral reasoning can be ascertained from the response to moral dilemmas or fictional short stories that describe situations in which a participant must make a moral decision. The participant is asked a systematic series of open-ended questions, like what they think the right course of action is, as well as justifications as to why certain actions are right or wrong.

### The present study

Since previous results have suggested that psychological distance is associated with differentiated sensitivity to the principles of justice (Engelmann et al., 2018), we hypothesize that the popular appeal of the proposal to decrease the APM in Brazil can be understood under the same framework. Thus, in the present work, we aim to verify whether the access to the reality of the youth judicial system, as a proxy to psychological distance, has a greater impact on the opinion on the reduction of the ACM. We also attempted to verify whether there is a distinct profile, in terms of both sociodemographic and moral development variables associated with being either in favor or opposed to reduction of the ACM.

## Materials and methods

### Participants

The research was approved by the Ethics and Research Committee with Humans of the Federal University of Para (UFPA) (approval #2.150.425). A total of 234 adult subjects participated in the study (77 in location 1 and 157 in location 2). Table 1 shows the sociodemographic characteristics of participants in both locations.

**TABLE 1:** Sociodemographic characteristics of participants.

Variables	Total sample *N* = 234	Location 1 *n* = 157	Location 2 *n* = 77	*P*-value^(1)^
	
		*n* (%)		
**Sex**				
Male	108 (46.15)	81 (51.59)	27 (35.06)	0.017*
Female	126 (53.85)	76 (48.41)	50 (69.94)	
**Age (years)**				
18–28	79 (33.77)	64 (40.77)	15 (19.48)	0.003**
29–39	58 (24.79)	32 (20.38)	26 (33.78)	
40–50	48 (20.51)	26 (16.56)	22 (28.57)	
51–59	25 (10.68)	16 (10.19)	9 (11.68)	
≥60	24 (10.25)	19 (12.10)	5 (6.49)	
**Marital status**				
Single	111 (47.45)	81 (51.60)	29 (37.66)	0.046*
Married	77 (32.91)	48 (30.58)	30 (38.98)	
Divorced	15 (6.41)	5 (3.18)	10 (12.98)	
Widower	4 (1.70)	3 (1.91)	1 (1.29)	
Stable union	26 (11.11)	19 (12.10)	7 (9.09)	
Not answered	1 (0.42)	1 (0.63)	0 (0.00)	
**Religion**				
Catholic	139 (59.42)	100 (63.71)	39 (50.66)	0.205
Protestant	51 (21.81)	30 (19.11)	21 (27.29)	
Spiritist	12 (5.12)	8 (5.09)	4 (5.19)	
Candomblé	1 (0.42)	0 (0.00)	1 (1.29)	
Other	6 (2.56)	4 (2.54)	2 (2.59)	
Without religion	19 (8.13)	13 (8.28)	6 (7.80)	
Atheist	4 (1.70)	2 (1.27)	2. (2.59)	
Not answered	2 (0.84)	0 (0.00)	2 (2.59)	
**Race (self-declared)**				
White	63 (26.92)	46 (29.30)	17 (22.08)	0.113
Black	15 (6.41)	7 (4.45)	8 (10.39)	
Yellow	5 (2.13)	2 (1.27)	3 (3.89)	
Brown	147 (62.84)	98 (62.44)	49 (63.64)	
Indigenous	0 (0.00)	0 (0.00)	0 (0.00)	
Not answered	4 (1.70)	4 (2.54)	0 (0.00)	
**Level of poverty**				
Non-existent (0%)	51 (21.80)	35 (22.29)	16 (20.77)	0.208
Low (1 to 25%)	43 (18.37)	31 (19.74)	12 (15.58)	
Medium (26–50%)	43 (18.37)	23 (14.65)	20 (25.98)	
High (51 to 100%)	97 (41.46)	68 (43.32)	29 (37.67)	
**Occupation**				
Intern	22 (9.40)	12 (7.64)	10 (12.99)	< 0.001**
Private employee	35 (14.96)	32 (20.38)	3 (3.89)	
Self-employed	39 (16.66)	26 (16.56)	13 (16.89)	
Public employee	71 (30.35)	29 (18.47)	42 (54.55)	
Others	27 (11.53)	22 (14.01)	5 (6.49)	
Not answered	40 (17.10)	36 (22.94)	4 (5.19)	
**Level of education**				
Fundamental (incomplete)	14 (5.98)	8 (5.09)	6 (7.80)	0.491
Fundamental (complete)	4 (1.70)	4 (2.54)	0 (0.00)	
Medium (incomplete)	14 (5.98)	10 (6.36)	4 (5.20)	
Medium (complete)	52 (22.23)	39 (24.85)	13 (16.88)	
Higher (incomplete)	55 (23.51)	36 (22.94)	19 (24.67)	
Higher (complete)	50 (21.37)	31 (19.75)	19 (24.67)	
Specialization	31 (13.25)	18 (11.47)	13 (16.88)	
Masters	9 (3.85)	6 (3.82)	3 (3.90)	
Doctorate	2 (0.85)	2 (1.27)	0 (0.00)	
Not reported	3 (1.28)	3 (1.91)	0 (0.00)	
**Family income** (MW)				
Below 2	56 (23.93)	37 (23.56)	19 (24.67)	0.133
Up to 2	26 (11.11)	21 (13.38)	5 (6.50)	
2 to 4	51 (21.80)	37 (23.56)	14 (18.18)	
4 to 10	45 (19.24)	25 (15.93)	20 (25.98)	
10–20	33 (14.10)	19 (12.11)	14 (18.18)	
>20	12 (5.12)	8 (5.09)	4 (5.20)	
Not reported	11 (4.70)	10 (6.37)	1 (1.29)	
**Family composition**				
Nuclear	120 (51.29)	79 (50.32)	41 (53.25)	0.608
Mononuclear	25 (10.68)	15 (9.56)	10 (12.99)	
Extended nuclear	30 (12.83)	23 (14.66)	7 (9.09)	
Extended mononuclear	7 (2.99)	6 (3.82)	1 (1.30)	
Live alone	18 (7.69)	10 (6.36)	8 (10.39)	
Other	5 (2.13)	4 (2.54)	1 (1.29)	
Not reported	29 (12.39)	20 (12.74)	9 (11.69)	
**Victim of juvenile violence**				
Yes	132 (56.41)	91 (58.96)	41 (53.24)	0.494
Not	102 (43.59)	66 (42.04)	36 (46.75)	

MW, minimum wage.

^1^Pearson’s chi-square (p value < 0.05).

**Values highly significant; *Significant values.

### Instruments

Subjects answered a sociodemographic questionnaire composed of 14 questions (gender, age group, marital status, religion, race, level of slums, occupation, level of education, family income, and family structure). The subjects also responded to three questions: “Have you heard about the proposal for decreasing the age of penal majority?” (YES/NO), “Do you agree with the proposal for decreasing the age of penal majority?” (AGREE, INDIFFERENT, DISAGREE), “Have you ever been the victim of juvenile crime?” (YES/NO).

The level of moral competence of subjects was assessed with a moral dilemma featuring adolescents in conflict with the law and based on the Moral Competence Test (MCT) designed by Lind (2000) according to Kohlberg’s theory of moral development (Kohlberg, 1974; Mathes, 2019). The moral dilemma was based on a short story about the occurrence of several cell phone thefts committed by an underage teenager and about the possibility, or not, of arresting him after the store owner tampered with the date of the footage and handed it over to the police. Right after reading the dilemma, the participant was instructed to respond with his opinion on the store owner’s decision. Responses were obtained in a Likert format from −3 to + 3, ranging from strong disagreement to strong agreement. Then, in the same vein, 12 arguments, six of which were favorable and six against the protagonist’s action, were also answered, in a Likert format (−4 to + 4), ranging from strong disagreement to strong agreement.

### Experimental procedure

Data were collected in two public places in the city of Belem (PA) through individualized interviews: a public square located at the center of Belem (PA) (Batista Campos Square, Location 1), and the Juvenile Court of Justice (Location 2). The choice of Location 1 is justified by the fact that it is a place where there is a large circulation of people with different economic and demographic profiles. Subjects in Location 2 (1st, 2nd and 3rd Courts of Childhood and Youth of the city of Belém), on the other hand, are judges, lawyers, psychologists, and social workers who interact with juvenile transgressors and their families in their daily routine.

Participants were selected by convenience at both locations. At location 1, the interviews took place on weekends and were conducted in the open air. At Location 2, the interviews took place during weekdays and were conducted in a private room. The places should represent opposite contexts in terms of psychological distance to the targets (adolescent transgressors), with Location 1 high and Location 2 low on average. At both locations, researchers first explained the purpose of the research and participants signed an informed consent form. Each participant had up to 60 min to complete the tasks.

### Data analysis

The stage of moral development (Lind, 2011) and C Index (Moral Competency Level) (Lind, 2000) were calculated for each subject and the C index was averaged by study location. The difference between the C Index was considered “high” when larger than five points and “very high” when larger than 10 (Lind, 2000). We performed data analysis with conventional statistical tests. Pearson’s chi-square test (χ^2^) was used to evaluate possible associations between categorical variables with statistical significance less than 0.05. Then, we performed a multivariate analysis by multimodal logistic regression for socioeconomic and sociodemographic characteristics, preference for stages and opinion on the reduction of APM (agree, indifferent, and disagree) as dependent variables.

## Results

Table 1 shows that the two locations differed on gender (*p* = 0.017), age group (*p* = 0.003), marital status (*p* = 0.046) and occupation (*p* < 0.001). Most people interviewed at Location 1 were employees in private companies (20.38%), male (51.59%), 18–28 years old (40.77%), single (51.60%). In contrast, Location 2 interviewees were mostly public employees (54.55%), female (69.94%), 29–39 years old (33.78%), married (38.98%). There was no statistically significant difference between the two groups regarding the other variables (Table 1).

Regarding the preference for stages of moral development, the subjects sampled in Location 2 had preference for lower stages (38.96% for stage 1), while in Location 1 the preference was for higher stages (17.19% for stage 6) (χ^2^ = 30.01, df = 1, *p* = 0.021). Subjects at Location 2 had a lower level of moral competence (3.97 points) than the public at Location 1 (14.29 points), according to the average C Index that evaluates moral competence, with a “very high” difference (10.32 points).

**FIGURE 1:**
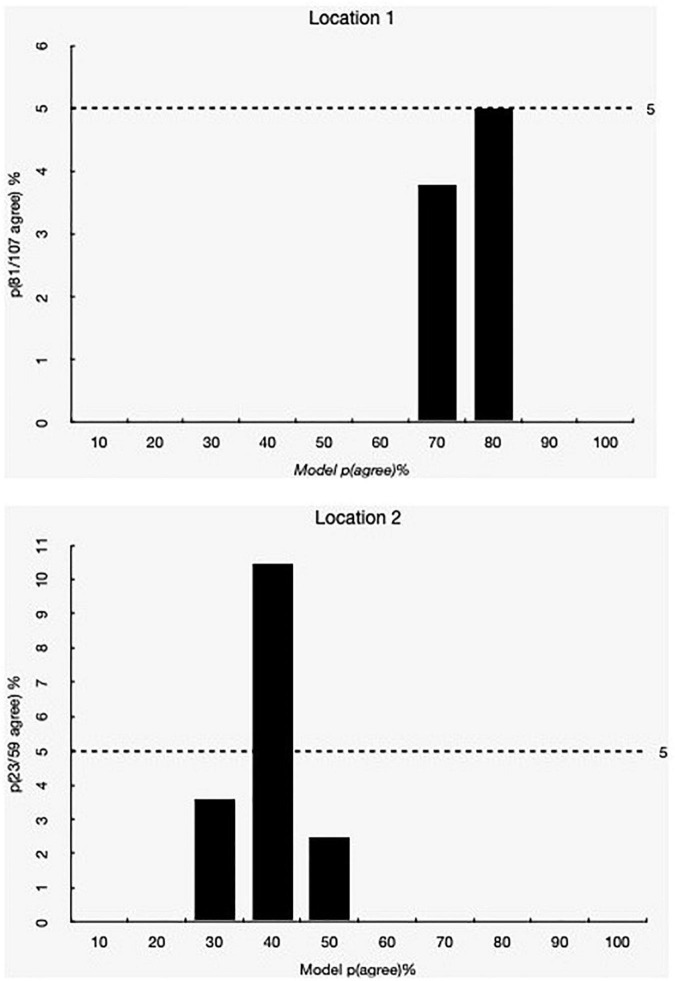
Probability mass of binomial distribution of agreement with the reduction of the APM at locations 1 **(A)** and 2 **(B)**.

Table 1 shows the participants’ opinions about the proposed reduction in APM according to location. Most people interviewed at Location 1 were in favor of the proposal (81, 51.6%), unlike those interviewed at Location 2, where the majority is against it (36, 46.81%) (χ^2^ = 24.535, df = 2, *p* < 0.001). The binomial probability mass function (Ross, 2020) of the agreement to the question “do you agree with the proposal for decreasing the age of criminal responsibility?” shows that the probability of agreement at location 1 is less than 80%, while in Location 2 is 30% (Figure 1).

The typical profile of those in favor of decreasing the APM are men (60, 57.7%) (χ^2^ = 14,336, df = 2, *p* < 0.001), married (43, 42.2%) (χ^2^ = 13,612, df = 6, *p* = 0.034), catholic (65, 62.5%) (χ^2^ = 9,413, df = 8, *p* = 0.042), living in a neighborhood with a high slum level (48, 46.2%) (χ^2^ = 6,754, df = 6, *p* = 0.049), with only basic education (47, 45.6%) (χ^2^ = 15,219, df = 4, *p* = 0.004), and earning less than 2 minimum wages (34, 34.3%) (χ^2^ = 11,920, df = 10, *p* = 0.005).

There was also an association between the preference for stages of moral development and opinion on decreasing the APM for the participants from Location 2: there was a preference for stage 1 among those who disagreed and for stage 6 among those who agreed (χ^2^ = 20,665, df = 10, *p* = 0.024).

The multivariate analysis by multimodal logistic regression identified two variables that are associated with the difference in opinion on the reduction of APM: Local (χ^2^ = 16,232, df = 2, *p* = 0.001) and sex (χ^2^ = 8,828, df = 2, *p* = 0.012) (Table 2). A further analysis showed that 64.4% of women in Location 2 disagreed with the reduction in the criminal majority (χ^2^ = 18,344, df = 2; *p* < 0,001) (Table 3).

**TABLE 2:** Multimodal logistic regression for the “agree” opinion on reduction of the age of penal majority.

Variables	β	Wald	Significant	OR	IC 95%
Local	1.492	13.083	0.000**	4.445	1.981–9.974
Sex	1.210	8.281	0.004**	3.352	1.471–7.641
Age (years)	–0.062	0.118	0.731	0.940	0.661–1.336
Marital status	0.296	0.644	0.422	1.345	0.652–2.772
Religion	–0.280	3.598	0.058	0.756	0.566–1.009
Race (self-declared)	0.252	3.083	0.079	1.286	0.971–1.704
Level of poverty	0.196	1.252	0.263	1.217	0.863–1.716
Occupation	–0.041	0.097	0.755	0.959	0.739–1.245
Level of education	0.064	0.022	0.883	1.067	0.451–2.521
Family Income (MW)	–0.228	2.879	0.090	0.796	0.612–1.036
Family composition	0.654	2.610	0.106	1.923	0.870–4.253
Victim of juvenile violence	–0.288	0.540	0.462	0.750	0.348–1.615
Levels of reasoning	0.116	1.080	0.299	1.123	0.902–1.399

**Highly significant.

**TABLE 3:** Crossover between variables sex, location and opinion on the reduction of APM.

Variables		Total sample *N* = 234	Opinion			*P*-value^(1)^
			Agree (*n* = 104)	Indiferent (*n* = 68)	Disagree (*n* = 62)	
		
		*N* (%)		*n* (%)		
Male	Location 1	81 (75.0)	47 (78.3)	24 (77.4)	10 (58.8)	0.244
	Location 2	27 (25.0)	13 (21.7)	7 (22.6)	7 (41.2)	
	Total	108 (100.0)	60 (100.0)	31 (100.0)	17 (100.0)	
Female	Location 1	76 (60.3)	34 (77.3)	26 (70.3)	16 (35.6)	0.001**
	Location 2	50 (39.7)	10 (22.7)	11 (29.7)	29 (64.4)	
	Total	126 (100.0)	44 (100.0)	37 (100.0)	45 (100.0)	

^1^Pearson’s chi-square (p value < 0.05).

**Highly significant;.

## Discussion

According to the Brazilian National Register of Adolescents in Conflict with the Law, in 2019, 189,000 adolescents were sentenced to socio-educational measures in the country, twice the number recorded in 2018 (96,000). The escalation of juvenile delinquency represented by those numbers and the spread of misinformation and fear about crime (Ambrey et al., 2014; Intravia, 2019), has increased the popular outcry for more severe and punitive measures for juvenile offenders. This led to several proposals of constitutional amendment to decrease the APR from 18 to 16 y.o. (Vavassori and Toneli, 2015). The first proposal from 1993 is based on the argument that due to greater access to information, the “discerning capability” of today’s youngsters is higher than in the 1940s when the APR was initially determined in Brazil. This reasoning is, together with the justification that “if they can vote they should be criminally imputable as well,” very popular with supporters of the reduction of the APR.

While juvenile transgressions receive a large share of attention (Pizarro et al., 2007), especially in populist discourse, the fact is adolescents are also a main target of violence. For instance, not only the two main causes of mortality among male adolescents are road injury and interpersonal violence, but mental disorders, including childhood behavioral, anxiety, and depressive disorders, are among the leading causes of morbidity among adolescents of both sexes and across age groups (Guthold et al., 2021). In Brazil (with data from only 18 of the 26 federal states), 29,512 adolescents aged 15–19 y.o. met intentional violent deaths, during 2016–2020, an average of 5,902.4 per year (UNICEF, 2021).

### Socio-cognitive considerations

The proposals for the decrease in the APR currently being considered by the Brazilian parliament runs against scientific evidence suggesting that the timespan of human adolescence is steadily increasing (Steinberg, 2014) and, if anything, the APR should also increase in order to protect and rehabilitate adolescent transgressors (Sawyer et al., 2018). Thus, in effect, such a measure will probably fail in meeting the objectives of fighting crime and violence.

We hypothesize that proposals for harsher juvenile justice legislation and the dismantling of the protections guaranteed to adolescents in conflict with the law results, in part, from a construal heuristic that is influenced by psychological distance to the problem and its social circumstances. Our findings give support to this hypothesis and show that subject’s agreement with the proposal to decrease the APR are correlated with moral development. Though morality is primarily a philosophical, rather than a behavioral, concept, it nonetheless informs decisions that have serious social implications. Differences in moral development in adults are not just differences in perception or comprehension of a situation.

Kohlberg proposes that at lower stages, as opposed to higher stages, morality is more subject to redefinition by specific context and by one’s social frame of reference and less by a fixed set of universal abstract moral principles. This is supported by our findings showing that subjects at lower stages of moral development tended to disagree with the proposal to decrease the APR. Individuals with lower levels of moral competence tend to advocate harsher punishment for transgressions while suppressing their moral judgment regarding their behavior. This can be explained by the greater reliance on cognitive heuristics associated with system 1 processes during moral judgments, viz-á-viz dual-process theory (Campbell and Kumar, 2012).

### Socio-demographic considerations

Our results also highlight the influence of socio-demographic factors, such as income, gender, marital status, religion, and education on opinion about the APM. For instance, subjects with lower income who live in neighborhoods with a high slum level tend to be in favor of the proposal while those with a higher income and who live in areas with low slum levels tend to disagree. This conflicting result could be explained by the widespread criminalization of “dangerous” peripherical neighborhoods in the media and an increased perception of their higher exposure to youth violence. The content of crime-related media is a determining factor in the perception of crime risk (Callanan, 2012) and may encourage individuals to be more punitive in their opinions. Regarding gender, our results show that men tend to be more favorable to the proposal of reducing the APM. Other studies also point to divergences between the sexes regarding decision-making and moral judgment (Capraro and Sippel, 2017; Acevedo-Triana et al., 2019). Both utilitarian and deontological rationalities founded in the notion of genetic and cultural co-evolution could explain the existing differences between the actions and the moral feelings of men and women. Studies have shown that there is a difference in moral assessment according to Efferson and Glenn (2018) and this could be explained by evolutionary pressure on reward pathways in the brain (Wilson et al., 2013; Soutschek et al., 2017). Gender differences in altruistic behaviors in humans show that women tend to be more equalitarian than men (Andreoni and Vesterlund, 2001) and could be less sensitive to construal imperatives.

As for marital status, while married people tend to agree with the decrease of the PAM, singles remain indifferent and divorced people disagree. This divergence in opinion may be related to the different familial experiences of these groups. Married people generally have more experience with other people depending on them, both materially and emotionally. This may lead them to feel more insecure regarding the prospect of urban violence which can affect not only themselves but those they care while being predisposed to be more punitive (Gopalkrishnan, 2018).

In terms of religion, Catholics tend to agree more with the proposal to decrease the APR while those without religion or atheists tend to disagree. Religion and notions of morality are deeply intertwined in human cultures (Purzycki et al., 2018). The notion of religion as a precondition to morality is largely prevalent in Brazil, where more than 83% of respondents in a multinational survey agree that morality is impossible without belief in god (Pew Research Center for the People and The Press, 2007). Thus, this majority is strongly influence by the perceived religious content of moral issues and usually adhere to a conservative worldview which is more intolerant on youth transgressions and argue for stronger punitive measures (Muncie, 2008).

Educational level significantly influences opinion on the APR. While those with only basic education tend to agree with the reduction of the APR, subjects with higher education stand with the opposite. This difference of opinion can be explained by the effect of knowledge on construal abstraction of events proposed by Kyung and coworkers (Kyung et al., 2014) that the more knowledge about an issue, the greater the possibility of contextual proximal influence on the opinion we form of people or events. Those with only basic education may prioritize basic ontogenetic principles in decision making, influenced, in most cases, by religion and family (Ho et al., 2015).

## Conclusion

Proposals for the reduction of the APR are motivated by a combination, among other factors, of fear of violence, distrust of juvenile rehabilitation/correctional programs, and a widespread misunderstanding of adolescent behavior. Though the central goal of rehabilitation is desistance of crime, most implementations put too much emphasis on the agency of the offender, leaving out societal responsibilities in ensuring adequate conditions for decreasing recidivism. Societal responsibilities are especially important regarding juvenile offenders, which are undergoing a dynamic process of cortical maturation which leaves them susceptible to impulsive behavior and increased vulnerability to peer-pressure. In general, especially after the advent of social media, society has adopted an increasingly punitive mentality, with people being easily canceled and condemned to social death. Thus, it does not seem surprising that populist punitive initiatives such as the reduction of the APM enjoy widespread support. This support is also motivated by a misguided perception of cognitive agency in adolescents, which is contrary to scientific findings regarding the adolescent mind (Steinberg, 2014). Most experts recommend that rehabilitative approaches combining the therapeutic and desistance paradigms seem to be more appropriate to dealing with adolescent transgressors and helping crime rates decrease (Droppelmann et al., 2022).

## Data availability statement

The raw data supporting the conclusions of this article will be made available by the authors under reasonable request.

## Ethics statement

The studies involving human participants were reviewed and approved by the Ethics and Research Committee with Humans of the Federal University of Pará (UFPA) (approval #2.150.425). The patients/participants provided their written informed consent to participate in this study.

## Author contributions

IC, IP, and AP designed the experiments and collected the data. IC, IP, KL, HM, PB, RM, and AP analyzed the data. IC, IP, RM, and AP wrote the manuscript. All authors contributed to the article and approved the submitted version.
